# Identification of *metE* as a Second Target of the sRNA scr5239 in *Streptomyces coelicolor*

**DOI:** 10.1371/journal.pone.0120147

**Published:** 2015-03-18

**Authors:** Michael-Paul Vockenhuber, Nona Heueis, Beatrix Suess

**Affiliations:** Department of Biology, Technical University Darmstadt, Darmstadt, Germany; Kunming University of Science and Technology, CHINA

## Abstract

While transcriptional regulation of the primary and secondary metabolism of the model organism *Streptomyces coelicolor* is well studied, little is still known about the role small noncoding RNAs (sRNAs) play in regulating gene expression in this organism. Here, we report the identification of a second target of the sRNA scr5239, an sRNA highly conserved in streptomycetes. The 159 nt long sRNA binds its target, the mRNA of the cobalamin independent methionine synthase *metE* (SCO0985), at the 5’ end of its open reading frame thereby repressing translation. We show that a high methionine level induces expression of scr5239 itself. This leads, in a negative feedback loop, to the repression of methionine biosynthesis. In contrast to the first reported target of this sRNA, the agarase *dagA*, this interaction seems to be conserved in a wide number of streptomycetes.

## Introduction

Small noncoding RNAs (sRNAs) represent a class of molecules that add a further level of posttranscriptional regulation to the regulatory networks of bacteria. They usually are not translated and most arise from the intergenic regions of the genome where they are transcribed from their own promoters. These often are induced by specific stress conditions or environmental and morphological changes, which leads to a very efficient and defined regulation of sRNA expression [1,2]. Most sRNAs act by binding directly to or nearby the translation initiation region of their target mRNAs thereby influencing the initial steps of translation [3]. Using different experimental approaches, sRNAs have been found in a vast number of bacteria and archaea [4–8]. Most knowledge about sRNAs, their targets as well as their regulatory interactions that is available today, however, has been gathered from Gram-negative bacteria such as *Escherichia coli* and *Salmonella typhimurium* [9,10].

Streptomycetes are Gram-positive, GC-rich, soil dwelling bacteria, with some existent marine isolates. The members of this large class of bacteria usually undergo extensive morphological changes throughout their development, starting from a single spore to filamentous mycelia and finally aerial hyphae. *Streptomyces coelicolor* is the model organism for this group of bacteria. It has a 8.7 Mb linear genome with approximately 8000 annotated genes and about 100 predicted or verified sRNA genes (reviewed in [11]).

The *S* *treptomyces* *c* *oelicolor* sRNA upstream of SCO5239: scr5239 was identified in a deep sequencing approach in 2011 [4]. It is one of only two noncoding RNAs in the genus *Streptomyces* where a target has been identified [12,13]. The sRNA is conserved in nearly two-thirds of the currently available *Streptomyces* genomes. Its secondary structure is that of five consecutive stem-loops, with the sequence of stem P4 being involved in the interaction with its first known target mRNA *dagA*. DagA is an extracellular agarase responsible for the first step of agar degradation, the cleavage of agar into the disaccharide neoagarobiose. However, while scr5239 is quite well conserved in Streptomycetes, *dagA* is not. This prompted us to screen for further targets of scr5239.

The small RNA scr5239 is constitutively expressed at a basal level throughout the developmental cycle. In this study we show, however, that its expression level does change depending on the availability of different amino acids like glutamine, glutamate and methionine. Methionine is an essential amino acid in all organisms. As a proteinogenic amino acid it is best known for its important role during the initiation of translation but it is also the key component of S-adenosyl methionine (SAM), the cells main donor of methyl groups [14]. Like most bacterial species, *S*. *coelicolor* possesses a biosynthetic pathway for methionine production. In general methionine is synthesized from aspartate via homoserine, serine, cysteine and homocysteine. A comprehensive review of methionine biosynthesis in bacteria was published recently [15]. The final step of methionine synthesis is the S-methylation of homocysteine. In *S*. *coelicolor* two independent non-homologous enzymes can carry out this reaction: The cobalamin-dependent methionine synthase MetH (SCO1657) and the cobalamin-independent MetE (SCO0985). In the MetH catalysed reaction cobalamin serves as a methyl acceptor and donor giving MetH a roughly 50-fold higher turnover rate then its functional sibling MetE [16,17]. The decision as to which enzyme is used depends on the availability of adenosylcobalamin (AdoCbl or coenzyme B12). The *metE* gene carries a B12-sensing riboswitch in its 5’UTR. Binding of coenzyme B12 to the riboswitch leads to the formation of a transcriptional terminator and repression of *metE* expression. In the absence of coenzyme B12 the *metE* mRNA is synthesized. It is unknown if and how *metH* expression is repressed in that case.

The second necessary input signal for *metE* expression is the feedback regulation by the methionine level in the cell that determines if there is a need for methionine synthesis. The exact nature and the members of this regulatory process, however, are not known [18]. In this work we show that, in the case of *metE*, methionine dependent repression of the synthase is achieved by binding of a small noncoding RNA to the *metE* mRNA.

## Methods

### Cultivation of *Streptomycetes*

108 spores of S. coelicolor M145 per 50 ml media were pre-germinated as described by Kieser et al. [19]. Cultures were grown in TSB (Becton-Dickinson) or Evans medium at 28°C under continuous shaking. Basic Evans medium contains: 0.25 mM CaCl_2_, 100 mM MOPS, 10 mM KCl, 2 mM Na_2_SO_4_, 2.5 mM citric acid, 1.25 mM MgCl_2_, 1 nM NaMoO_4_ and 5 ml trace element solution [40 mg ZnCl_2_, 200 mg FeCl_3_ x 6 H_2_O and 10 mg CuCl_2_ x 2 H_2_O, MnCl_2_ x 4 H_2_O, Na_2_B_4_O_7_ x 10 H_2_O and (NH_4_)_6_Mo_7_O_24_ x 4 H_2_O per 100 ml solution]. The pH was adjusted to 7.2 with NaOH and the volume was increased to 90% of the final volume. After autoclaving the C, N and P sources were added (125 mM glucose, 100 mM NaNO_3_ and 16 mM NaH_2_PO_4_ [12] Amino acid solutions were freshly prepared, filter sterilized and added after autoclaving the medium to the final concentration as indicated.

### Plasmid construction

Construction of the plasmids pUWL-scr5239+ (overexpression), pUWL-asscr5239 (knockdown) and pHDV_scr5239 (*in vitro* transcription) was described previously [12]. For *in vitro* transcription of the *metE* mRNA fragments M1 or M2 the respective fragment was amplified from chromosomal DNA (M1: 1,038,088–1,038,302 nt, M2: 1,038,088–1,038,245 nt). The PCR products were cloned into the plasmid pSP64 [20] using the restriction sites *Eco*RI and *Hind*III. The resulting plasmids were named pSP64_M1 and pSP64_M2. For cloning of the scr5239 *gusA* promoter fusion the region 100 nt upstream of the sRNAs transcription start site to 20 nt downstream of it (5,699,761 to 5,699,880 nt in the *S*. *coelicolor* genome) was amplified from chromosomal DNA. The PCR product was cloned into the plasmid pGusA [21] using the restriction sites *Bgl*II and *Acc*65I giving the plasmid pGusA_scr5239p. To create *metE* reporter gene fusions the M1/M2 fragments were PCR-amplified from the respective pSP64 variant and cloned into the plasmid pGusA using the restriction sites *Acc*56I and *Age*I. The *metH* 5’UTR and the first 30 nt of the ORF were PCR amplified from the genome (1,776,304–1,776,414 nt). The *metH* reporter gene fusions were then created as described for *metE*. Expression of the fusion protein and the control was driven by the constitutive promoter of the *ccpA* gene SCO4158 (4,576,135–4,576,184 nt). All oligonucleotide sequences can be found in supplementary S1 Table, plasmid maps are available upon request.

### Deletion of scr5239

To delete scr5239 including its promoter (5,699,776–5,700,019 nt), the 1,000 nt up- and downstream of the sRNA were PCR-amplified from the genome and fused by overlap PCR to flank a kanamycin (km) resistance cassette. The resulting fragment was cloned into the single *Xba*I site of the suicide plasmid pKCLP2 [22] carrying an apramycin (apr) resistance cassette giving pKCLP2_dscr5239. The plasmid was transferred into *S*. *coelicolor* M145 by intergeneric conjugation from *E*. *coli* ET12567/pUZ8002. scr5239 deletion mutants were identified by replica plating and screening for apr^S^/km^R^ clones. The deletion of scr5239 was confirmed by colony PCR, sequencing of the PCR product and northern blot (S2b Fig and S1 Table).

### RNA Isolation

Total RNA was isolated using the hot phenol method described by Mattatall *et al*. [23]. Five ml cultures were harvested and resuspended in 300 μl lysis buffer (10 mM sodium acetate, 150 mM sucrose, pH 4.8). Glass beads (200 μl, 0.4 mm diameter) and 300 μl hot phenol (65°C) were added. Cells were disrupted using a FastPrep-24 instrument (MP Biomedicals) for 5x 1 min at 6.5 m/s. After phenol/chlorophorm extraction and ethanol precipitation the RNA was resuspended in 500 μl ddH_2_O and the concentration was determined (usually 2–4 μg/μl). One hundred mg of total RNA were incubated with 30 U Turbo DNase (Ambion) for 1 h to remove residual DNA, subsequently precipitated and resuspended in 50 μl ddH_2_O. Usually a concentration of 1–1.5 μg/μl was yielded and 1 μg was quality checked on a 1% agarose gel.

### Northern blot analysis

Ten mg of total RNA were separated on 6% denaturing polyacrylamide (PAA) gels and transferred to a positively charged nylon membrane (Hybond N+, GE Healthcare) in a tank blotting device (Peqlab) at 4°C. Ten pmol oligonucleotide 1-E or 5S_short were radiolabeled at the 5’ end using 4 μl γ-^32^P-ATP (~ 1.6 pmol/μl, Hartmann-Analytik) and 1 μl T4 polynucleotide kinase (Roche) in the supplied buffer for 1 h at 37°C and purified with Illustra Microspin G-25 columns (GE Healthcare). Approximately 5 pmol of the labeled oligonucleotides (~ 300 kcpm/μl) were used as probe for one experiment. Signals were quantified by phosphoimaging using a Typhoon phosphoimager (GE Healthcare). Expression of scr5239 (probe: 1-E, see S1 Table) was normalized to the amount of 5S rRNA (probe: 5S_short, see S1 Table).

### *In vitro* transcription

For *in vitro* transcription the template plasmid pHDV_scr5239 or pSP64_M1 was linearized using the restriction enzyme *Hind*III. After phenol/chlorophorm extraction and isopropanol precipitation the linear plasmid was resuspended in ddH_2_O. Transcription was carried out over night at 37°C in a 2 ml reaction containing 200 μg linear plasmid, 25 mM magnesium acetate, 200 mM Tris-HCl pH 8.0, 20 mM DTT, 2 mM spermidine, 4 mM of each NTP and 2.5 μl T7 RNA polymerase (home-made). After the incubation 1 ml 0.5 M EDTA pH 8.0 was added to dissolve pyrophosphate. The mixture was subsequently precipitated, dissolved in 3 ml RNA loading dye (deionized formamide with 25 mM EDTA) and loaded onto a 6% denaturing PAA gel with 8 M urea. After the run, the transcribed RNA was visualized in the gel by UV-shadowing, excised and eluted overnight at 4°C in 300 mM sodium acetate pH 6.5. After ethanol precipitation the RNA was dissolved in ddH_2_O.

### Electrophoretic mobility shift assay to detect RNA-RNA complex formation

Ten pmol *in vitro* transcribed RNA were 5’ labeled with γ-^32^P-ATP. Per lane, 0.5 μM unlabelled scr5239 RNA mixed with 50 kcpm of the ^32^P-labeled scr5239 RNA were incubated with 7 or 14 μM unlabeled M1 RNA in a 10 μl reaction containing 10 mM Tris pH 7.0, 100 mM KCl, 10 mM MgCl_2_ and 1 μg yeast tRNA (Invitrogen) to prevent unspecific binding. After 15 min incubation at 28°C each sample was mixed with 5 μl 5x native loading buffer (50% glycerol, 0.2% bromophenol blue, 0.5x TB) and loaded onto a running 6% native PAA gel—precooled to 4°C—with 0.5x TB as running buffer. After the run, the gel was dried for 30 min at 80°C. Radioactivity was detected using a FUJI FLA-5000 Phosphoimager. For competition assays 10 μM of the oligonucleotide 1-E (S1 Table) were added prior to the incubation.

### Measurement of ß-glucuronidase activity

10^8^ pre-germinated spores of *S*. *coelicolor* strains were inoculated in 50 ml Evans medium containing amino acids as depicted. Mycelia were harvested after 72 h of cultivation, washed with gus-buffer (50 mM NaHPO_4_ pH 7.0, 0.1% Triton X100, 5 mM DTT) and centrifuged again. The subsequent disruption of the cells was done using 200 μl glass beads and a FastPrep-24 instrument (MP Biomedicals) for 6x 30 s at 6.0 m/s. Mycelial debris were removed by centrifugation at 4°C. Protein concentration of the supernatant was determined by Bradford assay [24]. For measurement of GusA activity 25–50 μg protein in a total volume of 750 μl gus-buffer was incubated at 37°C for 15 min. The reaction was started by the addition of 80 μl 200 mM *p*-nitrophenyl-β-D-glucuronide (Glycosynth) and stopped with 200 μl 1 M Na_2_CO_3_ upon appearance of a yellowish coloration. Absorption at 415 nm was measured and divided by the reaction time to calculate Gus activity [A_415_ min^-1^] and further divided by the amount of protein used to calculate specific Gus activity [A_415_ mg^-1^ min^-1^], which was used herein to represent Gus units (GU). All assays were conducted in triplicate and repeated at least once. Data are presented as the mean +/- standard deviation.

### SDS-PAGE and silver staining

10^8^ pre-germinated spores of *S*. *coelicolor* strains were inoculated in 200 ml TSB medium and grown at 28°C under continuous shaking. After 3 days, 50 ml samples were harvested, washed twice in Evans medium and resuspended in Evans medium containing amino acids as indicated. The strains were incubated as before for another 24 h, harvested and washed twice in 1x ZAP (50 mM NaCl, 50 mM Tris-HCl pH 8.0, 10% glycerol, 10 mM PMSF). Cell disruption was done as described for the GUS measurements only in 800 μl 1x ZAP. Fifty μg of crude extract were separated on a 20 x 20 cm 8% PAA SDS gel [25] overnight at 4°C and 40 mA. For silver staining the gel was fixed in 50% methanol, 12% acetic acid and 0.18% CH_2_O overnight. The next day, the gel was washed three times for 20 s in 50% ethanol. Next, it was incubated in solution 1 (0.5% Na_2_S_2_O_3_) for one min followed by three washing steps in ddH_2_O for 20 s each. After 20 min incubation in solution 2 (1.25% AgNO_3_, 0.18% CH_2_O), the gel again was washed three times for 20 s in ddH_2_O and finally developed in solution 3 (6% Na_2_CO_3_, 0.12% CH_2_O, 2% solution 1). Once the protein bands were clearly visible, the reaction was stopped by addition of acetic acid to a final concentration of ~ 10%.

Protein bands showing a differential expression pattern were excised using a clean scalpel. Protein identification was done in the mass-spec facility of the Institute of Pharmaceutical Chemistry, Goethe University Frankfurt, Germany.

## Results

The sRNA scr5239 can be found in approximately two-thirds of the available sequenced *Streptomyces* genomes. However the only validated target of scr5239, the agarase *dagA* is present merely in *S*. *coelicolor*. This led us to the hypothesis, that scr5239 had to have further—possibly more conserved—target mRNAs.

**Fig 1:**
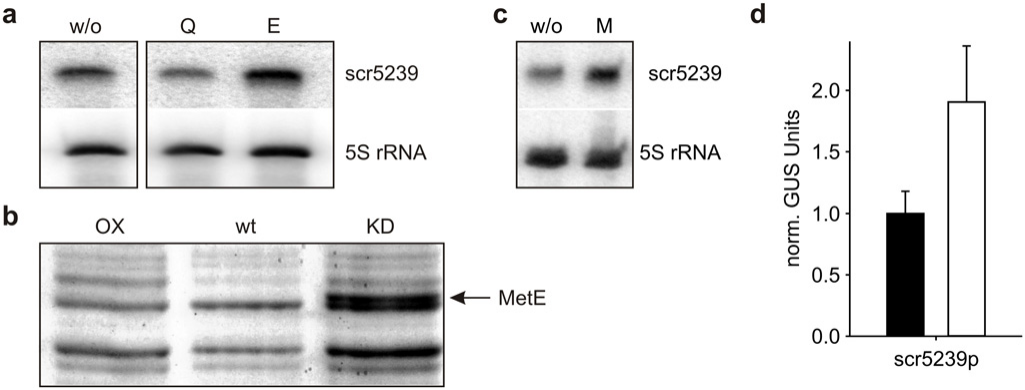
Expression analysis of scr5239 and its influence on the proteome. (**a**) Northern blot of scr5239 from wild type grown in minimal medium (w/o) or minimal medium supplemented with 1% glutamate (E) or 1% glutamine (Q). 5S rRNA was used as a loading control. (**b**) Silver stained SDS-PAGE of *S*. *coelicolor* wild type (wt), scr5239 overexpression (OX) and knockdown (KD) strain grown in minimal medium supplemented with glutamate. The upregulated protein appearing in the KD strain was identified by mass spectrometry to be the methionine synthase MetE. (**c**) Northern blot of scr5239 wild type grown in minimal medium (w/o) or minimal medium supplemented with 1% methionine (M). 5S rRNA was used as a loading control. (**d**) Reporter gene measurement of the scr5239 promoter fused to a *gusA* gene. Cells were grown as described in (**c**) without (black bar) or in presence of methionine (white bar).

Previously we have shown, that the expression of scr5239 decreases under nitrogen limiting conditions [12]. So, to get a lead on other targets of scr5239 we further investigated this phenotype. First, we analysed the expression of scr5239 in the presence of the key components of nitrogen storage, glutamine and glutamate. Fig. 1a shows a northern blot of *S*. *coelicolor* wild type cells grown in minimal medium supplemented with glutamine or glutamate. These two amino acids have a contrary effect; glutamine leads to a repression, glutamate to an induction of scr5239 expression (see S1 Fig for quantification of the northern blot). This finding prompted us to analyse the proteome of *S*. *coelicolor* under these conditions. Fig. 1b shows a gel slice, where overexpression (OX), wild type (wt) and knockdown (KD) strains of scr5239 [12] had been grown in the presence of glutamine. One prominent band specifically is induced in this medium in the scr5239 knockdown strain. This band was identified by mass spectrometry to be the B12-independent methionine synthase MetE (SCO0985).

MetE is one of two isoenzymes that catalyse the S-methylation of homocysteine to methionine. The other isoenzyme, MetH, is vitamin B12-dependent; it requires methylcobalamin (derived from B12) as a cofactor to catalyse the *de novo* formation of methionine. In bacteria that carry functionally equivalent B12-dependent and B12-independent enzymes, the activity of the B12-independent one often is regulated by a B12 riboswitch in the 5’UTR of the respective mRNA [26]. By comparing a large variety of bacterial genomes Vitreschak *et al*. [26] predicted the existence of a B12 riboswitch in the intergenic region 382–155 nt upstream of the *metE* ORF.

A transcriptome analysis of *S*. *coelicolor* grown on solid medium done in our lab supports this prediction. It places the *metE* transcription start point at 1,037,861 nt, 382 nt upstream of the GTG start codon (S2 Fig).

We addressed the question whether the expression of scr5239 was dependent on methionine. We cultivated the wild type in minimal medium +/- methionine and checked for sRNA expression in a northern blot. Quantification of the northern blots showed, that the presence of methionine in the medium leads to an almost 2-fold increase in the amount of scr5239 (Fig. 1c; p-value < 0.05). Fusion of the scr5239 promoter region to a *gusA* reporter gene led to the same increase showing that this effect is due to a higher transcription rate rather than a stabilization of the sRNA in the presence of methionine (Fig. 1d).

**Fig 2:**
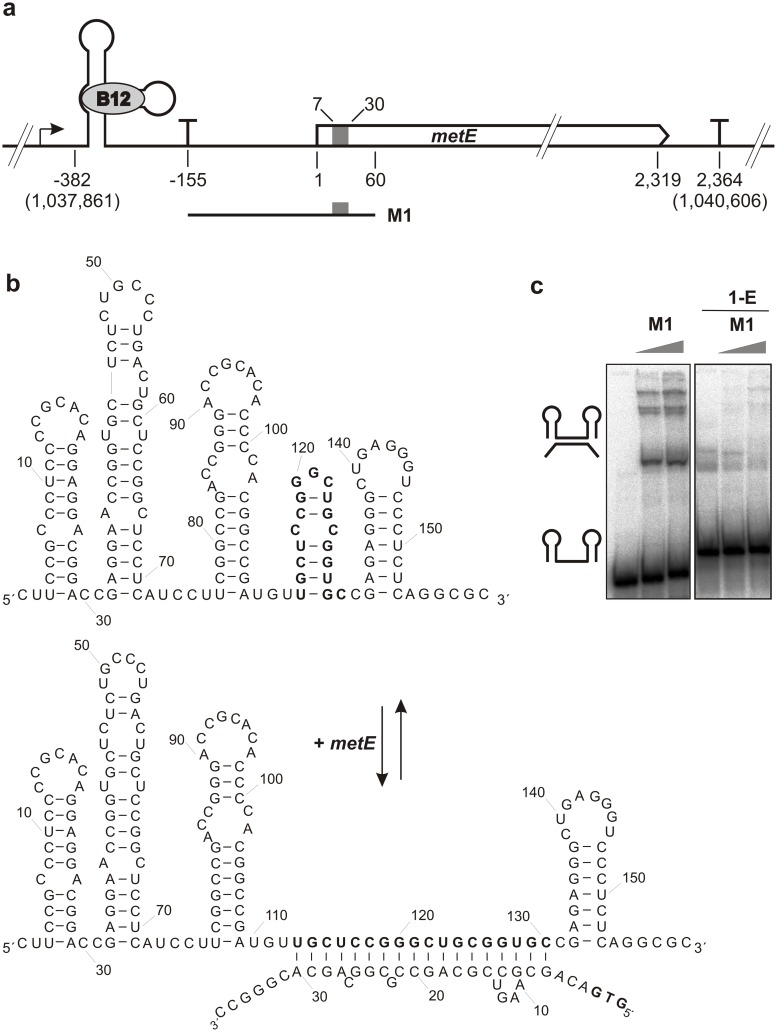
Genomic organisation of *metE* and its interaction with scr5239. (**a**) Overview of the *metE* gene (not to scale). Shown is the region of 1,037,861 to 1,040,606 nt of the *S*. *coelicolor* genome. The 382 nt long 5’UTR harbours a B12-dependent transcriptional riboswitch at its 5’ end. In the presence of B12 transcription terminates at position-155 (data not shown). The binding site of scr5239 is depicted as grey box, ranging from +7 to +30 in the *metE* ORF. Transcriptional terminators are depicted as T. The mRNA fragment M1 was used for *in vitro* analysis of the metE—scr5239 interaction. (**b**) 2D structure of scr5239 without *metE* mRNA bound to it. The binding site on scr5239 and the GTG start codon of *metE* are depicted in bold. (**c**) Mobility shift assay of radiolabeled scr5239 with the *metE* M1 fragment showing the interaction between the two RNAs (left panel). In the right panel scr5239 was preincubated with the DNA oligonucleotide 1-E blocking the binding site for *metE*.

In the next step, we tested whether scr5239 affects *metE* expression in a direct or indirect manner. Most sRNAs that repress translation of their targets bind them in or around the translation initiation region [1]. Using RNAhybrid [27] for sequence alignment we identified a potential scr5239 binding site on the *metE* mRNA spanning the area from nucleotide +7 to +30 in the ORF (Fig. 2a and b). On the sRNA side the binding site covers the nucleotides 114 to 130 which are almost exactly the same nucleotides as for the previously identified target *dagA* (nt 113 to 129) [12].

To test if there is a direct interaction between scr5239 and the *metE* mRNA we performed mobility shift assays with scr5239 and the *metE* mRNA. To omit possible interference of the B12 riboswitch during *in vitro* transcription or during refolding of the RNA after its purification, we used a 215 nt fragment of the *metE* mRNA (M1) covering the nucleotides-155 to +60. The mobility shift assay was performed using radiolabeled scr5239 RNA mixed with rising amounts of M1 RNA. Fig. 2c—left panel—shows that there is a direct and specific interaction between the two RNAs. The same effect can be seen when radiolabeled M1 RNA is titrated with scr5239 RNA (S3 Fig). To check, whether we predicted the correct binding site on the sRNA side, we performed another mobility shift assay where we added an excess of the DNA oligonucleotide 1-E thereby blocking the proposed binding side for access from M1 RNA (Fig. 2c, right panel). 1-E was able to abolish the interaction between scr5239 and M1, validating that the interaction site was predicted correctly. The multiple bands that can be seen in the assay arise from several conformations of the M1 RNA. In a native gel, the free M1 RNA adopts at least three visible conformations all of which are bound by scr5239 (see S3 Fig; first lane). We also verified, that scr5239 is able to bind to the full-length *metE* 5’UTR including the riboswitch. For that we performed another gel retardation assay using the complete *metE* 5’UTR spanning the nucleotides-382 to +60 (S4 Fig).

Fig. 2b shows the 2D structure of free scr5239 as validated by enzymatic and inline-probing as well as a model of the *metE*-scr5239 complex. Like for *dagA* the binding site of *metE* almost solely consisted of the nucleotides forming stem P4 that has to unfurl to allow for the interaction to take place.

In the next step, we wanted to confirm the interaction *in vivo*. The standard method to analyse sRNA-target interactions *in vivo* was developed for *E*. *coli* by Urban *et al*. in 2007 [28]. There, the sRNA of interest is expressed from a high-copy plasmid while a target-mRNA-GFP fusion is expressed from a low-copy plasmid to enhance the regulatory effect and the sensitivity of the system. Mutations in the sRNA and/or the mRNA fragment serve to validate the interaction. Since its publication, this system has been adapted for a wide range of organisms like *Sinorhizobium* [29] or *Vibrio* [30]. We redesigned this system for the use in *Streptomyces* to measure the scr5239-*metE* interaction. Due to the high intrinsic fluorescence of *S*. *coelicolor* we chose to use *gusA* instead of *gfp* as reporter gene. Further, we used the integrating plasmid pGusA to gain a reliable and stable copy-number of the reporter fusion. In addition, we decided to create a scr5239 deletion strain (Δscr5239), where the sRNA was replaced by a kanamycin resistance cassette (S5a and S5b Fig.). We chose to delete scr5239 because the previously used antisense-mediated knockdown strain [12] showed considerable variation in knockdown efficiency depending on the medium conditions used (data not shown).

**Fig 3:**
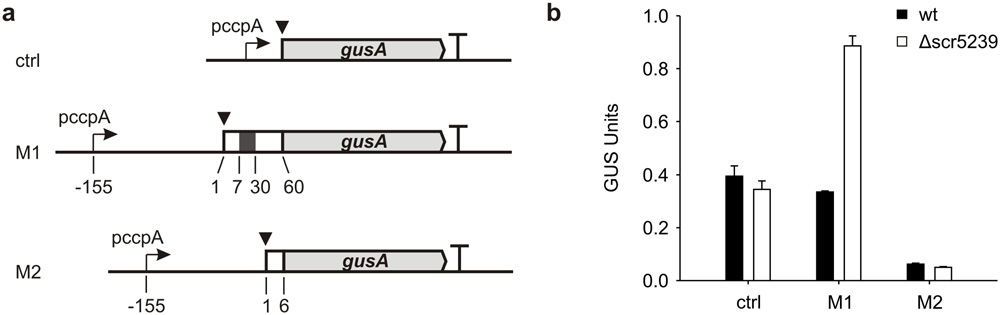
Reporter gene measurement of wt and Δscr5239 strain. (**a**) Scheme of the reporter gene constructs used. M1 covers the *metE* 5’UTR from-155 nt to +60 nt of the ORF. Translational fusions of the *metE* ORF to the *gusA* gene (light grey arrow) are depicted as white box. The scr5239 binding site is depicted as dark grey box. The respective start codon of the fusion protein is indicated by a black triangle. M2 covers the *metE* 5’UTR from-155 nt to +6 nt of the ORF, thereby lacking the scr5239 binding site. The control plasmid (ctrl) carries no *metE* sequences at all. Transcriptional terminators are depicted as T. Expression of all constructs is driven by the promoter of the *ccpA* gene (pccpA). (**b**) β-Glucuronidase assay of the constructs shown in (**a**). wt: wild type, Δscr5239: scr5239 deletion strain.

We used the same RNA sequence as for the mobility shift experiments for the *in vivo* measurement of the scr5239—*metE* interaction (construct M1). As a control we cloned a shorter fragment covering the same 5’UTR part but only the first six nucleotides of the *metE* ORF thereby omitting the scr5239 binding site (construct M2, Fig. 3a). As a second control we used the original pGusA plasmid without any *metE* fragments attached to the *gusA* mRNA. Measurement of reporter gene expression of *S*. *coelicolor* wt *vs*. Δscr5239 shows a 2.6-fold induction of *metE* expression in the deletion strain. This induction is lost in the M2 construct where the binding site is missing (Fig. 3b). The overall lower expression level of the M2 construct may be due to an altered translation efficiency and/or stability of the fusion protein. Scr5239 seems to regulate *metE* expression on the translational level as the mRNA abundance of *metE* does not change in the Δscr5239 strain compared to the wild type (S6 Fig).

These findings show that there is a negative regulation of *metE* expression by the methionine inducible sRNA scr5239. We also tested whether the second methionine synthase, *metH*, is regulated in the same way. A reporter gene fusion of the protein, however, showed no increase of MetH expression upon scr5239 deletion (S7 Fig).

## Discussion

In this study, we identified a second target of the sRNA scr5239 in *S*. *coelicolor*. This finding fits to the current understanding of sRNA function in bacteria: that sRNAs, like the eukaryotic microRNAs, usually do not regulate just one target but act as posttranscriptional control instances for whole networks of target genes [31]. One example for this is the *S*. *typhimurium* sRNA GcvB that is a major regulator of amino acid metabolism in this organism [32].

The binding site of scr5239 to its second target again is located within the ORF of the mRNA. In the best-studied examples, *E*. *coli* and *S*. *typhimurium*, it is known but rather unusual for an sRNA to bind its target in its ORF. The majority of sRNAs in these organisms regulate the expression of their targets by blocking access to the ribosomal binding site and/or start codon thereby hindering translation initiation. However, recent studies have shown that binding of an sRNA up to 5 codons downstream of the start codon still is sufficiently close to the translation initiation region to interfere with 30S subunit binding [3]. The binding site of scr5239 on the *metE* mRNA fits this rule as it covers the codons 3–10 (Fig. 2b). Interestingly, the binding site of scr5239 on *dagA* covers the codons 11–18 on the mRNA [12]. This supports our current proposal, that the regulation of *dagA*—a rather recent acquisition to the *S*. *coelicolor* genome—by scr5239 is still in the progress of being established.

In contrast to *dagA*, the *metE* gene including its binding site to scr5239 is highly conserved throughout the streptomycetes indicating that this regulatory interaction is much more widespread (S8 and S9 Figs). On the side of scr5239 a seven nucleotide core binding motif (GCUGCGG) is highly conserved in all homologs we identified so far. In about half the homologs this motif occurs—with one G to C variation—in tandem (GCUCCGG-GCUGCGG, see S8a Fig top five sequences). Being in the coding region of the *metE* gene, the binding site on the mRNA is even more highly conserved (S8b Fig). But a few variations occur: the core motif complementary to scr5239s is CCGCAGC. In *S*. *hygroscopicus* there is a silent A to G mutation that increases the possible binding site by three nucleotides (S9 Fig). An additional CGC insertion in the ORF three nucleotides downstream of the conserved motif also got integrated into the binding site on the sRNAs side.

Little is known about the regulation of amino acid metabolism in *Streptomyces*. To date, three conserved transcription factors involved in amino acid biosynthesis have been characterized: GlnR (glutamine, glutamate), ArgR (arginine) and NdgR (leucine, cysteine and methionine) [18,33,34]. NdgR has recently been shown to act as a transcriptional activator on at least two genes of the methionine synthesis system: *metH* and the methylenetetrahydrofolate reductase *metF* that catalyzes the second to last step of methionine synthesis. In this study, Kim *et al*. used the DNA sequences of the presumed promoter regions of the respective genes as bait and screened for NdgR binding. The *metE* probe, however, failed to capture any NdgR even though they used the whole intergenic region upstream of *metE* as bait. Kim *et al*. identified two possible binding motifs for NdgR within the probes that were captured by the protein. However, both of them are absent in the intergenic region upstream of the *metE* gene. This implies, that the two enzymes MetE and MetH are regulated by different means although they perform the same reaction: MetH expression is dependent on the presence of the transcriptional activator NdgR. The expression of MetE, on the other hand, depends both, on the absence of coenzyme B12 and of the sRNA scr5239.

**Fig 4:**
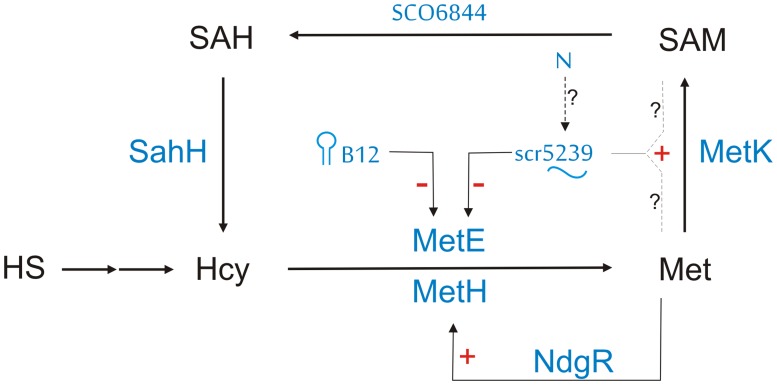
Model of the regulation of the methionine biosynthesis in *S*. *coelicolor*. Methionine is synthesised from homoserin (HS) via homocystein. Two methionine synthases, MetH and MetE, catalyse the final step. MetH which uses adenosylcobalamin as methyl donor catalyses the reaction with high turnover rate when coenzyme B12 is present. In the absence of B12, a B12-dependent riboswitch allows transcription of *metE* mRNA leading to increased methionine production by MetE. High methionine concentration results in feed back regulation of both MetH and MetE. MetH is controlled by the transcriptional regulator NdgR [18]. In addition, high methionin concentration also induces the expression of scr5239, which in turn represses *metE* translation. A possible link between nitrogen and methionine metabolism via the sRNA is speculative.

Fig. 4 shows a model of our current understanding of the role scr5239 plays in the regulation of *metE*. If coenzyme B12 is available the B12 dependent methionine synthase MetH is expressed while transcription of *metE* is terminated by the B12 riboswitch in its 5’UTR. If B12 is scarce, transcription of the *metE* mRNA is permitted and MetE is expressed. It is still unknown, if and how MetH expression is downregulated under these conditions. NdgR activates its transcription upon nutrient downshift [18], but there is no data showing if the availability of coenzyme B12 influences the activity NdgR and/or MetH.

Expression of MetE leads to an increased methionine level, which—by a yet unknown mechanism—induces the expression of scr5239. The sRNA then represses the translation of the *metE* mRNA thereby reducing methionine synthesis.

The S-methylation of homocysteine is the final step in methionine biosynthesis. While there are several alternative ways leading to homocysteine, this last step is highly conserved. The two methionine synthases MetH and MetE can be found in parallel in a large number of bacteria, although there are species that carry one but not the other [15]. The transcription of *metE* is regulated by a B12 riboswitch in its 5’UTR in most bacteria. This allows the cell to make the decision if coenzyme B12 is available and if therefore the expression of a cobalamin-independent methionine synthase is necessary. Some bacteria, like *Bacillus clausii*, additionally have a SAM riboswitch in tandem to the B12 riboswitch in their *metE* 5’UTR. Also the expression of *metH* and *metK* is controlled by a SAM riboswitch in this organism. This setup generates a feedback inhibition where high SAM levels directly repress the expression of these three enzymes [35]. Streptomycetes lack a SAM riboswitch in *metE* (also in *metH* and *metK*), therefore we propose that here the conserved sRNA scr5239 takes over its regulatory function: to give a feedback about the cells methionine (and SAM) supply.

Nature has come up with diverse solutions to tackle the problem of multiple signal input. In *B*. *subtilis*, the *glmS* riboswitch probably represents the simplest solution. The ligand binding pocket of the aptamer domain not only binds intracellular Glucosamine-6-phosphate as an activating agent. It also binds Glucose-6-phosphate, which acts as an inhibitor of ribozyme activity. The riboswitch, thus, integrates metabolite information to sense the overall metabolic state of the cell [36]. A second solitary element that senses and integrates two different input signals is the adenine riboswitch encoded by the *add* gene from *Vibrio vulnificus*. This translational regulatory element senses temperature and ligand concentration to confer efficient regulation over the range of physiologically relevant temperatures, allowing gene expression control to adapt to the differing temperatures experienced in the free-living and in the infectious environments [37]. As already discussed above, in *B*. *clausii*, two different riboswitches respond independently to SAM and vitamin B12, respectively. This system’s output fits a Boolean NOR gate, because transcription is terminated in the presence of only one or both ligands [35]. In the work presented here, not two riboswitches but a riboswitch combined with a regulatory small RNA mediate the input of the two signals. A combination of sRNAs and riboswitches regulation was also reported for SroA and SroG, two sRNA processed out of a riboswitch [38] or, even more intriguingly, two riboswitches which respond to SAM in *Listeria monocytogenes* which control the expression of the virulence regulator PrfA act as sRNA themselves [39]. On the contrary, a recent report describes a B12 riboswitch that is part of the sRNA EutX controlling ethanolamin utilization in *Enterococcus faecalis*. This riboswitch can lead to premature termination of the sRNA-target interaction therefore regulating the sRNA function. Obviously there are plenty of possibilities to perform multiple input integration, however to our knowledge; *metE* is the first example of an sRNA regulating gene expression in combination with a riboswitch.

What is the connection of the regulation of methionine synthesis to the glutamine/glutamate complex? Glutamine acts as nitrogen storage in the cell. Feeding the cells with glutamine shifts the balance towards nitrogen surplus and leads to repression of scr5239 expression (Fig. 1a). It is not clear, why methionine production would have to be upregulated under these conditions. Generally, nitrogen limitation is regarded as the ‘normal’ state for bacteria growing in soil [40,41]. This would be represented by a high glutamate over glutamine ratio leading to an induction of scr5239 and thereby a repression of methionine synthesis. A good nitrogen supply, however, can induce a period of fast growth where protein synthesis and thereby the demand for amino acids increases. Therefore scr5239 regulation possibly connects the nitrogen supply status of the cell to amino acid synthesis.

## Supporting Information

S1 Fig Quantification of the northern blot shown in Fig. 1a. Scr5239 signal was normalized to the 5S rRNA loading control.
(TIF)

S2 Fig RNAseq data of the *metE* mRNA. RNA was prepared at five different time points from *S*. *coelicolor* M145 grown on solid medium throughout one developmental cycle. The differentially coloured bars represent the sequencing reads from each time point. The transcription start point of *metE* was identified at position 1,037,861 nt. Transcription termination by the B12 riboswitch occurs at a terminator structure at 1,038,093 nt (155 nt upstream of the *metE* start codon).
(TIF)

S3 Fig Gel retardation assay of radiolabelled M1 RNA with increasing amounts of scr5239. Under native conditions the M1 RNA forms at least three different conformations (see first lane). Yet all of them seem to bind scr5239.
(TIF)

S4 Fig Gel retardation assay of scr5239 and *metE* mRNA fragments. M1: *metE* fragment M1 covering-155 to + 60 nt of the mRNA. FL: full-length 5’UTR of *metE* covering-382 to +60 nt of the mRNA. CE: 1 μg crude extract of *S*. *coelicolor* wild type. The full-length 5’UTR of *metE* is bound by scr5239 in a similar amount as the M1 fragment. Crude extract was added with the aim to increase binding efficiency by supplementing necessary protein(s). This did, however, in this setup not lead to an improved binding.
(TIF)

S5 Fig Deletion of scr5239. (**a**) Overview of the deletion strategy. The scr5239 gene including its promoter was replaced with a kanamycin resistance cassette by homologous recombination (-84 to +159 nt). The upstream gene SCO5238 has a putative terminator structure 20 nt upstream of the beginning of the deletion. The downstream gene SCO5239 gives rise to a leaderless transcript starting with the first nt of the start codon (unpublished data). (**b**) Northern blot validating the successful deletion of scr5239. The deletion was also confirmed by colony PCR of the mutant and subsequent sequencing of the PCR product (not shown).
(TIF)

S6 Fig Northern blot of *metE* mRNA from the wild type and the Δscr5239 strain. 16S rRNA was used as a loading control.
(TIF)

S7 Fig *metH* expression is not dependent of scr5239. **(a)** RNAseq results of the *metH* gene (see S1 Fig). The *metH* gene has a 84 nt long 5’UTR starting at 1,776,417. **(b)** Cloned reporter fusion of *metH* to *gusA* used to measure a possible regulation by scr5239. The whole 5’UTR including the first 30 nt of the *metH* ORF where used. T represents the artificial terminator 3’ of the *gusA* ORF that ensures efficient transcription termination. **(c)** RNAhybrid predicted one possible binding site for scr5239 at-27 to-53 of the *metH* 5’UTR. The conserved core motif used for binding *metE*, however, is only partially present (green box, compare S4b Fig). **(d)** Reporter gene measurement of the *metH* fusion protein. Deletion of scr5239 does not affect the expression of MetH:GusA.
(TIF)

S8 Fig Conservation of scr5239 binding site. **(a)** Sequence alignment of scr5239 from eight Streptomyces species. The nucleotides used for binding *metE* in *S*. *coelicolor* are indicated in blue (top lane). Nucleotides predicted to be involved in *metE* binding in the other Streptomycetes are also indicated in blue. The highly conserved core binding motif is boxed in green. **(b)** Sequence alignment of the *metE* coding region. Colour coding as in (a). The top four sequences of scr5239 and *metE* are identical in their binding sites. For a detailed view of binding site variations see S9 Fig.
(TIF)

S9 Fig Detailed view of binding site variations. Predicted binding mode of scr5239 to *metE* in *S*. *coelicolor* (sco), *S*. *griseus* (sgr), *S*. *venezuelae* (sve), *S*. *bingchenggensis* (sbi), and *S*. *hygroscopicus* (shy). Mutations in the sequences of the sRNA or *metE* are given in red, the start codon in bold.
(TIF)

S1 Table Oligonucleotides used in this study. (DOCX)
