# Supplementary figures and images for: Identification of metE as a Second Target of the sRNA scr5239 in Streptomyces coelicolor
Source: PLoS One. 2015 Mar 18;10(3):e0120147. doi: 10.1371/journal.pone.0120147 (PMC4365011; doi:10.1371/journal.pone.0120147)

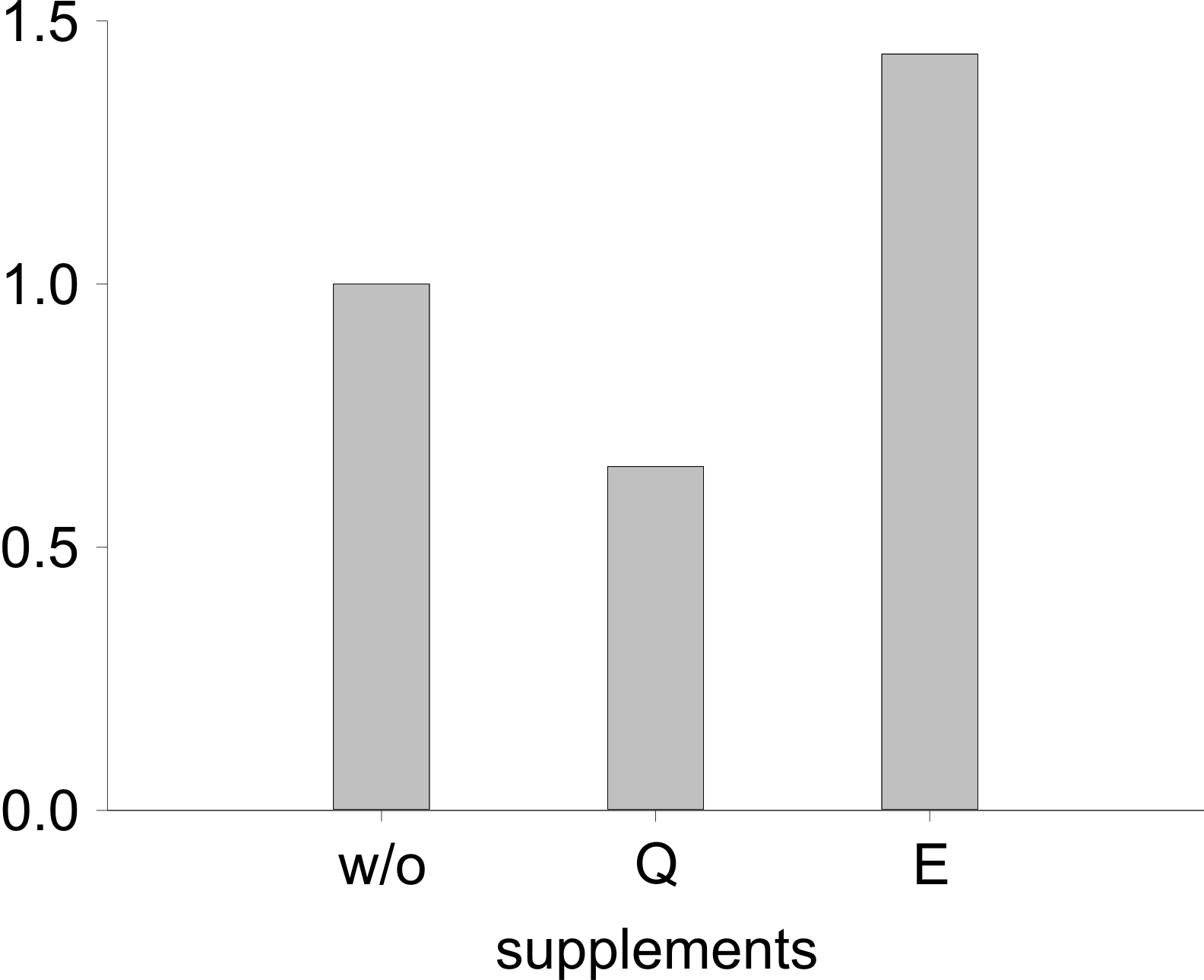

Supplement: S1 Fig — Scr5239 signal was normalized to the 5S rRNA loading control. (TIF) [file pone.0120147.s001.tif]

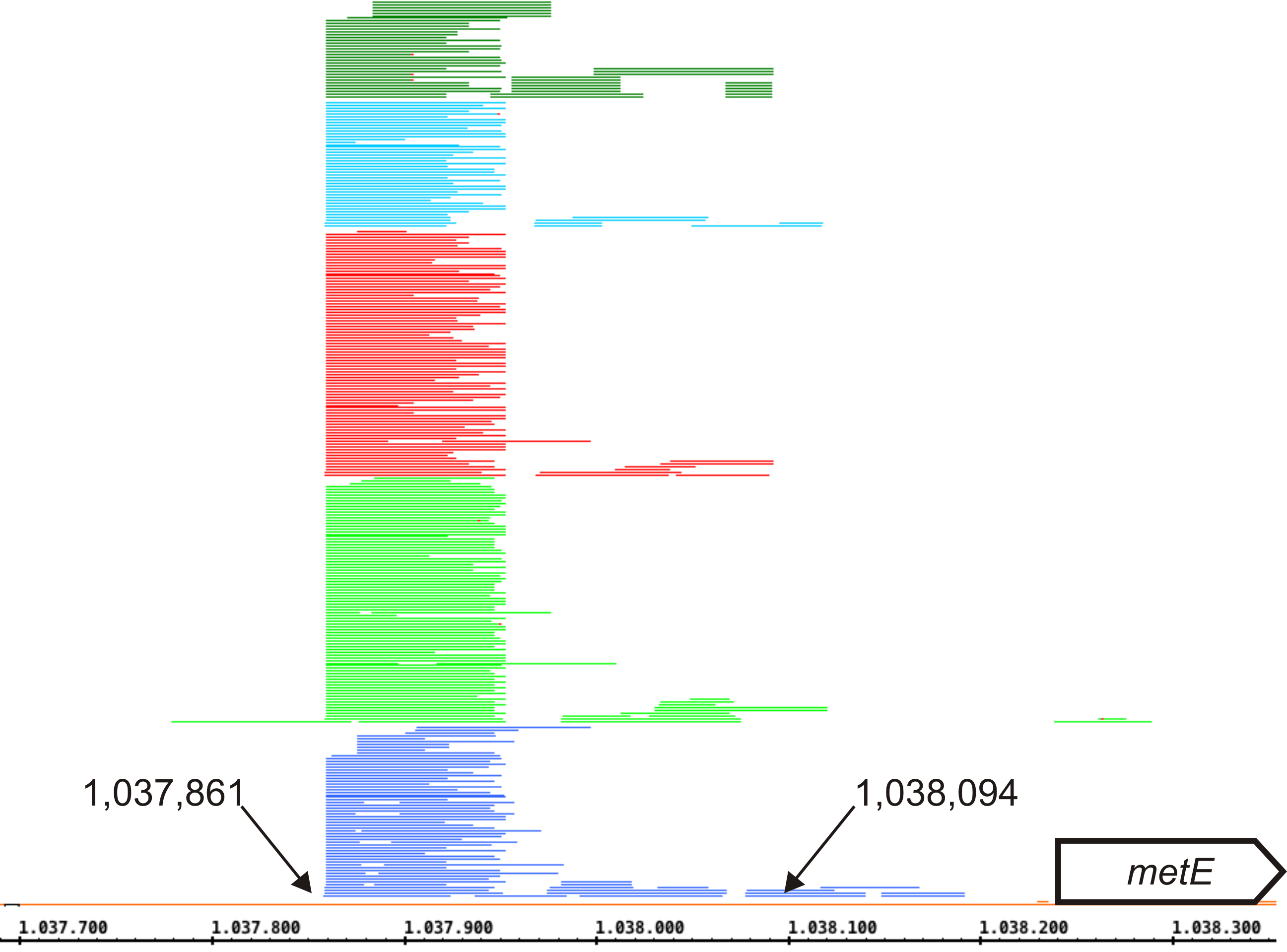

Supplement: S2 Fig — RNA was prepared at five different time points from S. coelicolor M145 grown on solid medium throughout one developmental cycle. The differentially coloured bars represent the sequencing reads from each time point. The transcription start point of metE was identified at position 1,037,861 nt. Transcription termination by the B12 riboswitch occurs at a terminator structure at 1,038,093 nt (155 nt upstream of the metE start codon). (TIF) [file pone.0120147.s002.tif]

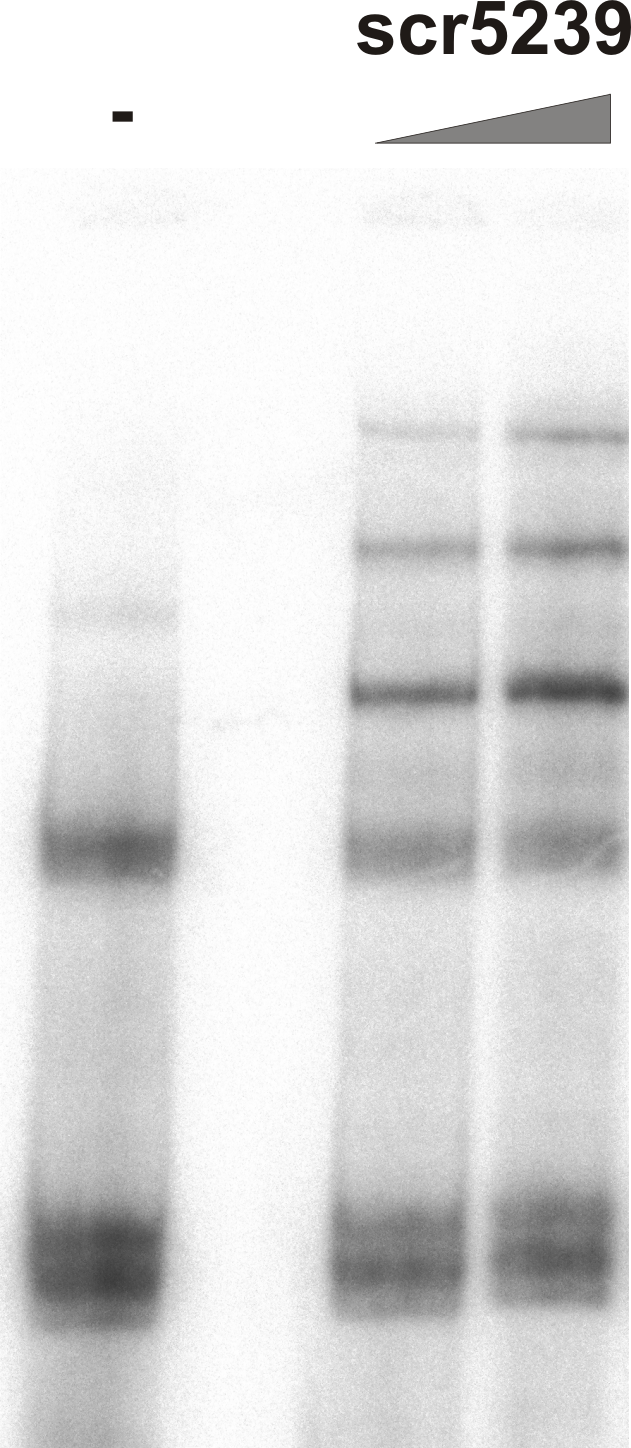

Supplement: S3 Fig — Under native conditions the M1 RNA forms at least three different conformations (see first lane). Yet all of them seem to bind scr5239. (TIF) [file pone.0120147.s003.tif]

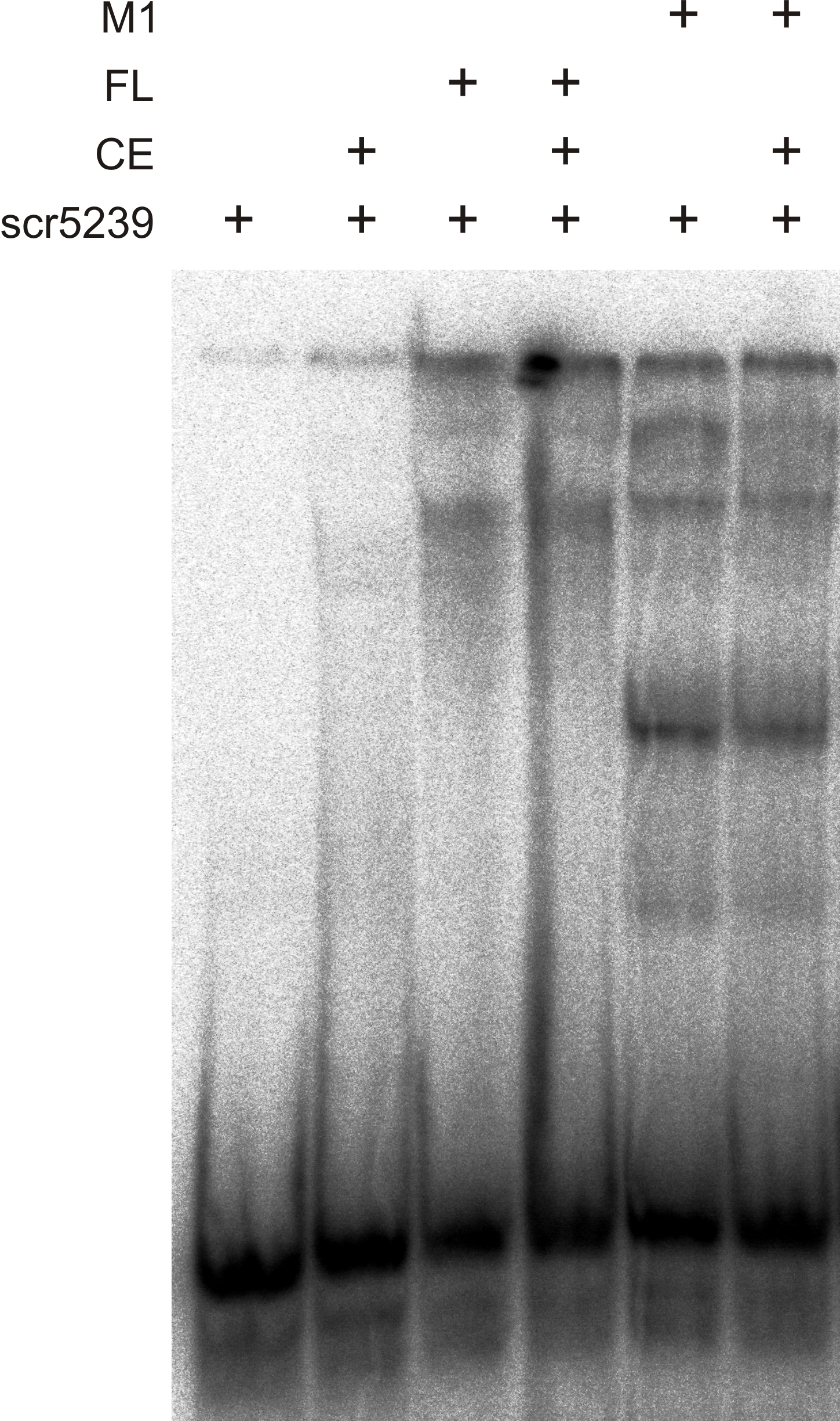

Supplement: S4 Fig — M1: metE fragment M1 covering-155 to + 60 nt of the mRNA. FL: full-length 5’UTR of metE covering-382 to +60 nt of the mRNA. CE: 1 μg crude extract of S. coelicolor wild type. The full-length 5’UTR of metE is bound by scr5239 in a similar amount as the M1 fragment. Crude extract was added with the aim to increase binding efficiency by supplementing necessary protein(s). This did, however, in this setup not lead to an improved binding. (TIF) [file pone.0120147.s004.tif]

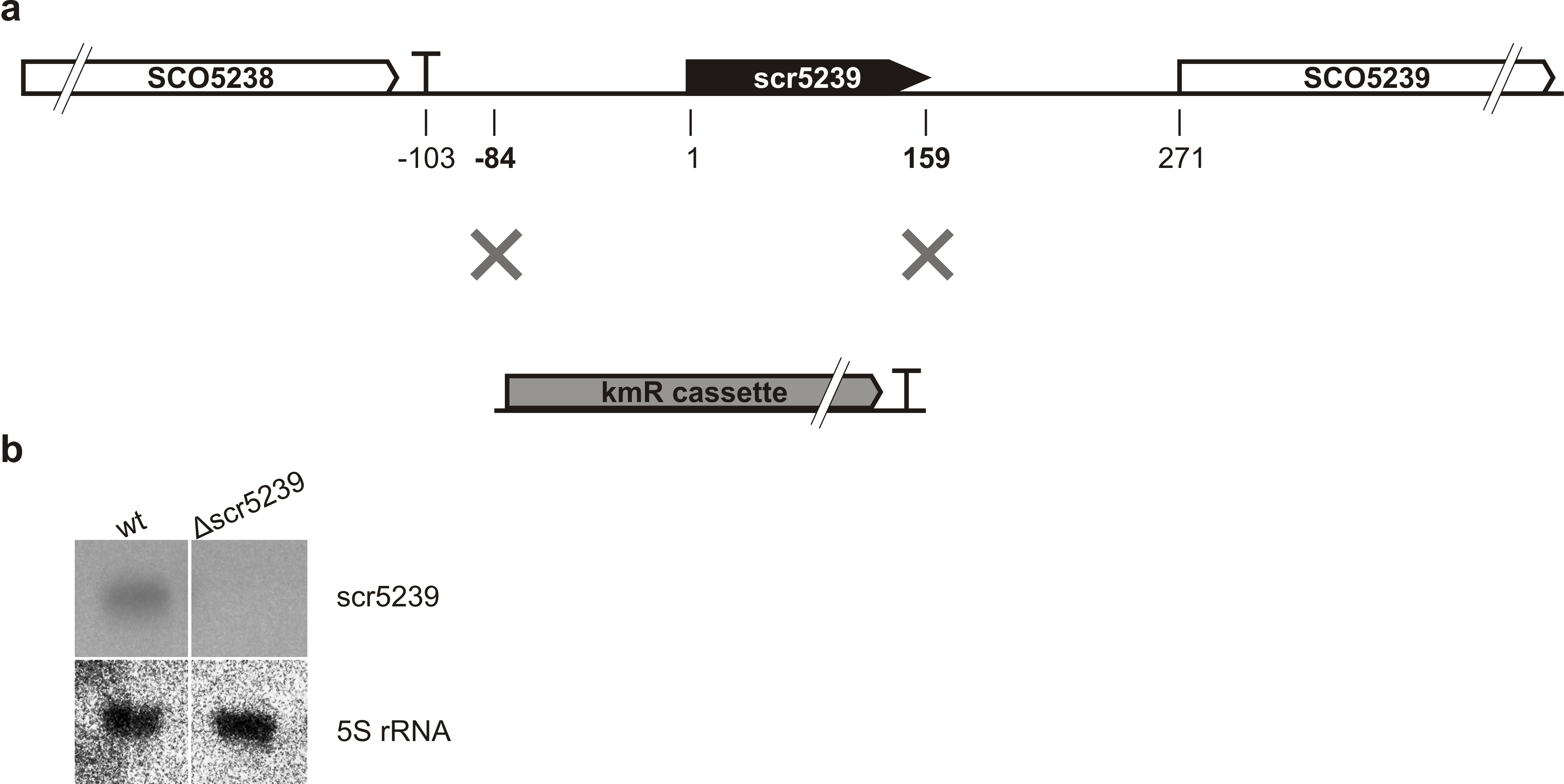

Supplement: S5 Fig — (a) Overview of the deletion strategy. The scr5239 gene including its promoter was replaced with a kanamycin resistance cassette by homologous recombination (-84 to +159 nt). The upstream gene SCO5238 has a putative terminator structure 20 nt upstream of the beginning of the deletion. The downstream gene SCO5239 gives rise to a leaderless transcript starting with the first nt of the start codon (unpublished data). (b) Northern blot validating the successful deletion of scr5239. The deletion was also confirmed by colony PCR of the mutant and subsequent sequencing of the PCR product (not shown). (TIF) [file pone.0120147.s005.tif]

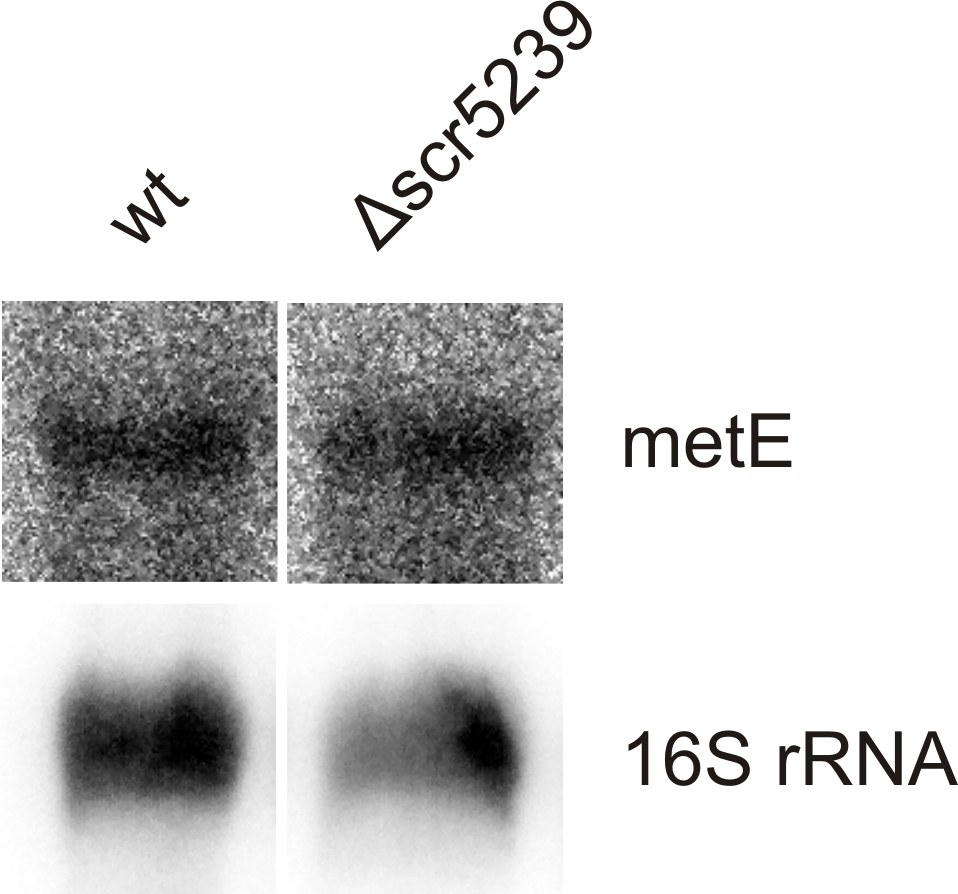

Supplement: S6 Fig — 16S rRNA was used as a loading control. (TIF) [file pone.0120147.s006.tif]

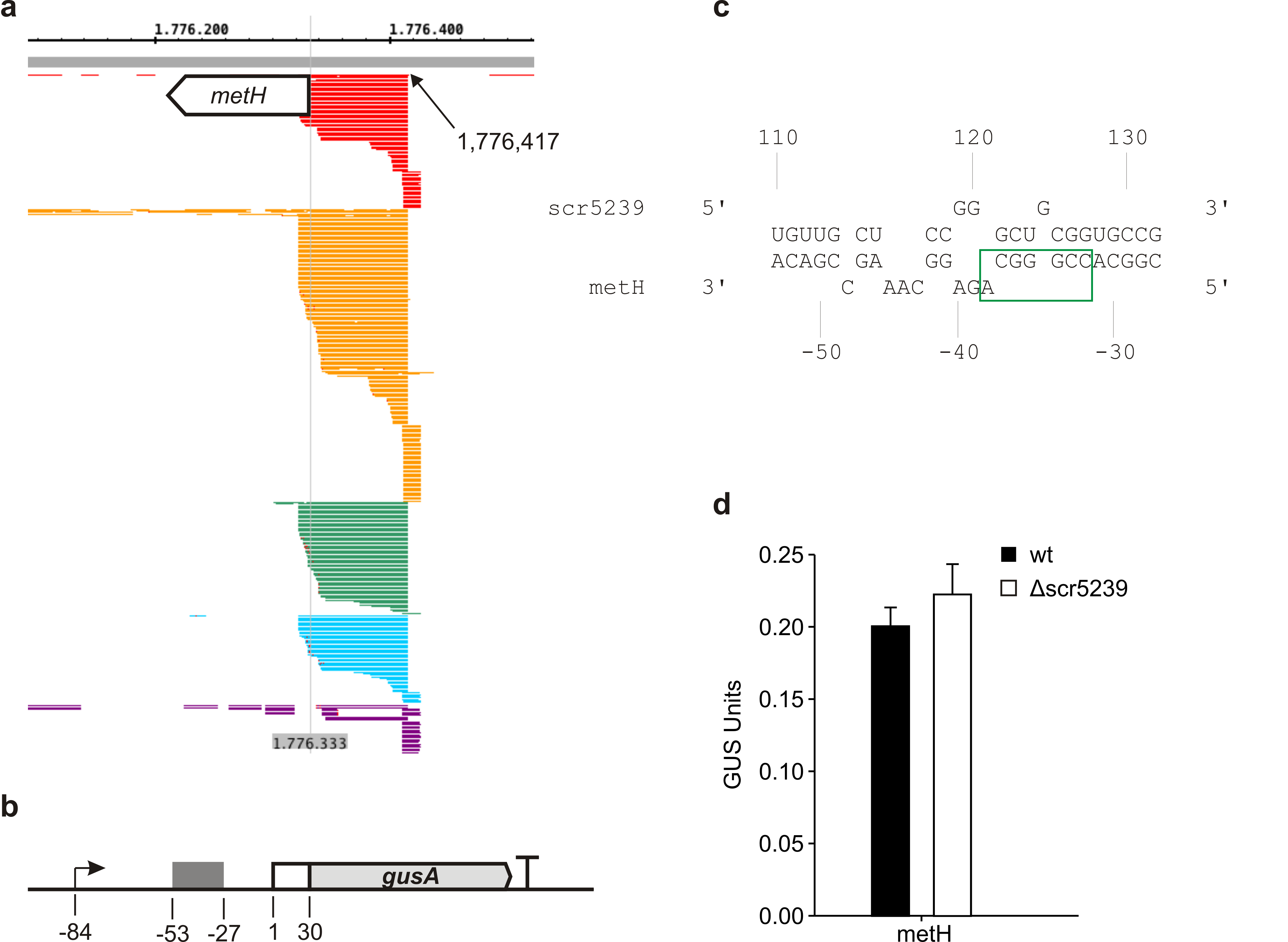

Supplement: S7 Fig — (a) RNAseq results of the metH gene (see S1 Fig). The metH gene has a 84 nt long 5’UTR starting at 1,776,417. (b) Cloned reporter fusion of metH to gusA used to measure a possible regulation by scr5239. The whole 5’UTR including the first 30 nt of the metH ORF where used. T represents the artificial terminator 3’ of the gusA ORF that ensures efficient transcription termination. (c) RNAhybrid predicted one possible binding site for scr5239 at-27 to-53 of the metH 5’UTR. The conserved core motif used for binding metE, however, is only partially present (green box, compare S4b Fig). (d) Reporter gene measurement of the metH fusion protein. Deletion of scr5239 does not affect the expression of MetH:GusA. (TIF) [file pone.0120147.s007.tif]

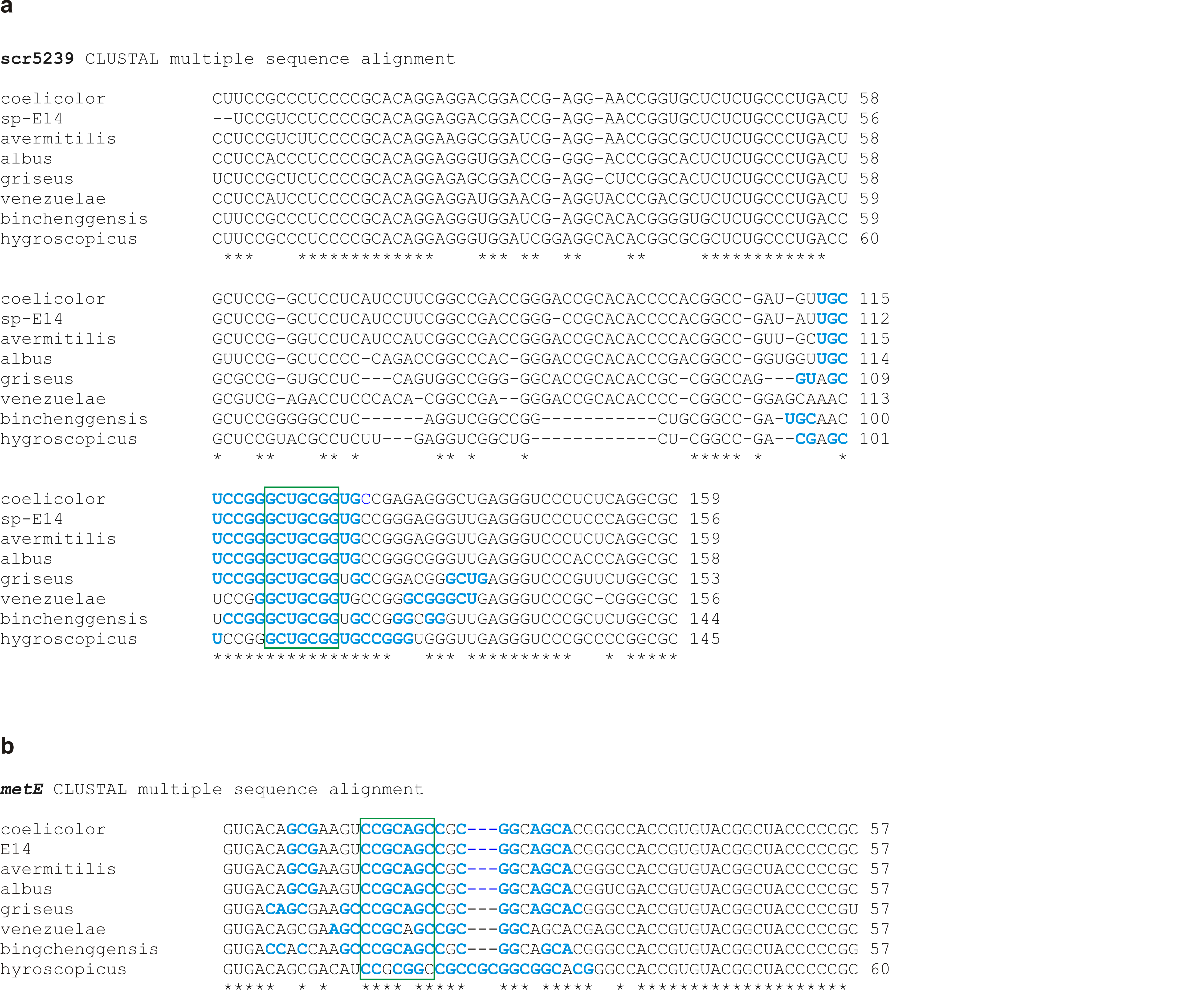

Supplement: S8 Fig — (a) Sequence alignment of scr5239 from eight Streptomyces species. The nucleotides used for binding metE in S. coelicolor are indicated in blue (top lane). Nucleotides predicted to be involved in metE binding in the other Streptomycetes are also indicated in blue. The highly conserved core binding motif is boxed in green. (b) Sequence alignment of the metE coding region. Colour coding as in (a). The top four sequences of scr5239 and metE are identical in their binding sites. For a detailed view of binding site variations see S9 Fig. (TIF) [file pone.0120147.s008.tif]

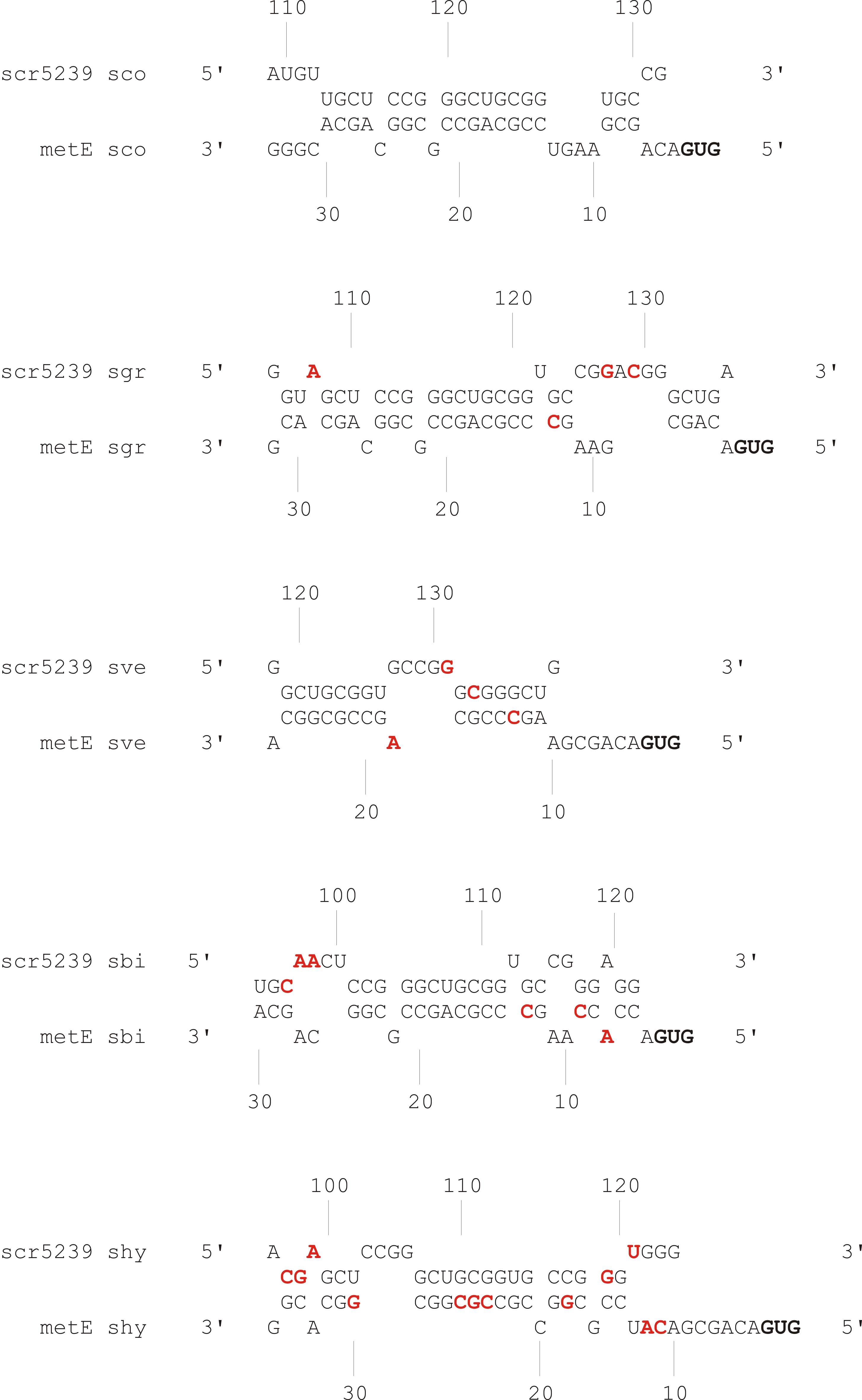

Supplement: S9 Fig — Predicted binding mode of scr5239 to metE in S. coelicolor (sco), S. griseus (sgr), S. venezuelae (sve), S. bingchenggensis (sbi), and S. hygroscopicus (shy). Mutations in the sequences of the sRNA or metE are given in red, the start codon in bold. (TIF) [file pone.0120147.s009.tif]
