# Supplementary material for: Identification of metE as a Second Target of the sRNA scr5239 in Streptomyces coelicolor
Source: PLoS One. 2015 Mar 18;10(3):e0120147. doi: 10.1371/journal.pone.0120147 (PMC4365011; doi:10.1371/journal.pone.0120147)
Supplement: S1 Table — (DOCX) [file pone.0120147.s010.docx]

**Table S1: Oligonucleotides used in this study**

| **name** | **sequence 5' -> 3'** | **application** |
| --- | --- | --- |
| 1-E | CGCAGCCCGGAGCAACAT | Northern probe scr5239 & EMSA |
| 5S_short | CGAAATGTAACCGGGCGTTT | Northern probe 5S rRNA |
| Cob-155_fwd | GATTACGAATTCCCAAGTAATACGACTCACTATAGGGACATCCCGGTGACCTCGAGG | cloning of M1 in pSP64 |
| Cob+60_rev | GAATACAAGCTTCTGGCGGGGGTAGCCGTACA |  |
| Psc1_f | ATCAGATCTCAACAACAAACAACGTCCACC | cloning of pGusA_scr5239p |
| Psc1_r | AAGCTTGGTACCCTGTGCGGGGAGGGCGGAA |  |
| up_f | GTCGACTCTAGAATCGCGGCGACCGTCTCCTT | deletion of scr5239 |
| upkm_r | TTGCGTTTTCCCTTGTCCAGAACCGTGTCCATGCGGTGGA |  |
| upkm_f | TCCACCGCATGGACACGGTTCTGGACAAGGGAAAACGCAA |  |
| downkm_r | GGAAGCACCCGCGGGCCGCGCCGAAGCCCAACCTTTCATA |  |
| downkm_f | TATGAAAGGTTGGGCTTCGGCGCGGCCCGCGGGTGCTTCC |  |
| down_r | GGCCGCGGATCCTCCCGGATGGTGGCGTCCTT |  |
| metE7_f | AGCGGGTACCACATCCCGGTGACCTCGAGGGCGGGT | cloning of pGusA_M1/M2 |
| metE1_r | CCGCAGACCGGTCTGGCGGGGGTAGCCGTACA | cloning of pGusA_M1 |
| metE11_r | CGAGACCGGTTGTCACGGAGATCTCCTTCG | cloning of pGusA_M2 |
| metH1_f | TGTAGCGGTACCGTGCGTTCTCCCCGGGACGT | cloning of pGusA_metH |
| metH1_r | CCGCAGACCGGTGGCGGGCGGGGTGGATGGCGA |  |
